# Taking the body off the mind: Decreased functional connectivity between somatomotor and default‐mode networks following Floatation‐REST

**DOI:** 10.1002/hbm.25429

**Published:** 2021-04-09

**Authors:** Obada Al Zoubi, Masaya Misaki, Jerzy Bodurka, Rayus Kuplicki, Colleen Wohlrab, William A. Schoenhals, Hazem H. Refai, Sahib S. Khalsa, Murray B. Stein, Martin P. Paulus, Justin S. Feinstein

**Affiliations:** ^1^ Laureate Institute for Brain Research Tulsa Oklahoma USA; ^2^ University of Oklahoma Tulsa Oklahoma USA; ^3^ Harvard Medical School Boston Massachusetts USA; ^4^ University of Tulsa Tulsa Oklahoma USA; ^5^ University of California San Diego San Diego California USA

**Keywords:** consciousness, default‐mode network, fMRI, insula, posterior cingulate, resting‐state, self, somatosensory

## Abstract

Floatation‐Reduced Environmental Stimulation Therapy (REST) is a procedure that reduces stimulation of the human nervous system by minimizing sensory signals from visual, auditory, olfactory, gustatory, thermal, tactile, vestibular, gravitational, and proprioceptive channels, in addition to minimizing musculoskeletal movement and speech. Initial research has found that Floatation‐REST can elicit short‐term reductions in anxiety, depression, and pain, yet little is known about the brain networks impacted by the intervention. This study represents the first functional neuroimaging investigation of Floatation‐REST, and we utilized a data‐driven exploratory analysis to determine whether the intervention leads to altered patterns of resting‐state functional connectivity (rsFC). Healthy participants underwent functional magnetic resonance imaging (fMRI) before and after 90 min of Floatation‐REST or a control condition that entailed resting supine in a zero‐gravity chair for an equivalent amount of time. Multivariate Distance Matrix Regression (MDMR), a statistically‐stringent whole‐brain searchlight approach, guided subsequent seed‐based connectivity analyses of the resting‐state fMRI data. MDMR identified peak clusters of rsFC change between the pre‐ and post‐float fMRI, revealing significant decreases in rsFC both within and between posterior hubs of the default‐mode network (DMN) and a large swath of cortical tissue encompassing the primary and secondary somatomotor cortices extending into the posterior insula. The control condition, an active form of REST, showed a similar pattern of reduced rsFC. Thus, reduced stimulation of the nervous system appears to be reflected by reduced rsFC within the brain networks most responsible for creating and mapping our sense of self.

## Graphical abstract

This study represents the first functional neuroimaging investigation of Floatation‐Reduced Environmental Stimulation Therapy (REST), an intervention that minimizes stimulation of the human nervous system. Utilizing a data‐driven exploratory analysis and an active control condition, this study found that Floatation‐REST elicited a consistent pattern of decreased resting‐state functional connectivity within and between posterior hubs of the default‐mode network and a large swath of cortical tissue centered around the somatosensory cortices.

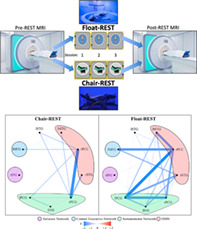

## 1 INTRODUCTION

Modern society and its evergrowing reliance on digital technology have exposed the human nervous system to an unprecedented level of sensory stimulation. While the capacity to respond and interact with external sensory stimuli constitutes a vital function of the brain, the ability to focus on one's self and disengage from external stimulation can be just as vital, but is far less studied (Suedfeld & Kristeller, 1982), and can be quite difficult for most to accomplish on their own, even over short periods (Wilson et al., 2014). Floatation‐Reduced Environmental Stimulation Therapy (REST) aims to effectively disengage the nervous system from sensory stimulation by creating a controlled environment which reduces most forms of external sensory input (including auditory, visual, olfactory, gustatory, tactile, thermal, vestibular, gravitational, and proprioceptive) while also minimizing musculoskeletal movement and speech (see Supporting Information for more details). The acute effect of this intervention appears to be one of heightened interoceptive awareness for cardiorespiratory sensations in the context of physiological relaxation, including reductions in blood pressure and muscle tension (Feinstein, Khalsa, Yeh, Al Zoubi, et al., 2018; Turner, Gerard, Hyland, Nieland, & Fine, 1993; Turner & Fine, 1983; Van Dierendonck & Te Nijenhuis, 2005). Subjectively, Floatation‐REST has been shown to elicit short‐term decreases in states of negative affect (e.g., anxiety, depression, and pain) and increases in states of positive affect (e.g., feeling refreshed, serene, and relaxed) (Feinstein, Khalsa, Yeh, Wohlrab, et al., 2018; Forgays & Belinson, 1986; Jacobs, Heilbronner, & Stanley, 1984; Kjellgren, Sundequist, Norlander, & Archer, 2001). To date, no neuroimaging studies have explored the neural effects of Floatation‐REST, and in general, little is known about how the nervous system responds following a prolonged period of reduced sensory and motor stimulation.

The default‐mode network (DMN) is thought to reflect the brain's intrinsic mental activity during periods of wakeful rest (Raichle, 2015; Raichle et al., 2001), making it a prime candidate for being impacted by the prolonged period of REST conferred by floatation. The DMN is comprised of two core regions: an anterior hub in the medial prefrontal cortex (mPFC) and a posterior hub in the posterior cingulate cortex (PCC) and precuneus. Beyond these hubs, the DMN also includes lateral flanks located near the temporoparietal junction in addition to regions within the medial temporal lobe (Andrews‐Hanna, Reidler, Sepulcre, Poulin, & Buckner, 2010). During nonactive tasks (e.g., while fixated on a baseline cross), there is a significant increase in resting‐state functional connectivity (rsFC) within and between hubs of the DMN. In line with this notion, the DMN is maximally active during times when external sensory stimulation is minimized and the mind is free to wander and reflect on the self. The inverse is also true such that DMN activity is rapidly suppressed whenever one is actively engaged with the outside world and attending to stimuli from the external environment. Over the past two decades, the observed increases in DMN rsFC during states of passive rest while one is conscious and awake has proven to be one of the most reliable and robust findings to emerge from the functional neuroimaging literature (Raichle, 2015; Termenon, Jaillard, Delon‐Martin, & Achard, 2016; Whitfield‐Gabrieli & Ford, 2012).

Decreases in DMN rsFC appear to be a common feature underlying altered states of consciousness, such as when falling asleep (Vallat, Meunier, Nicolas, & Ruby, 2019; Wang, Ong, Patanaik, Zhou, & Chee, 2016), or being deprived of sleep (De Havas, Parimal, Soon, & Chee, 2012), or while under anesthesia (Huang, Zhang, Wu, Mashour, & Hudetz, 2020) or subanesthetic doses of ketamine (Scheidegger et al., 2012). A similar pattern emerges under the acute influence of psychedelic drugs like ayahuasca (Palhano‐Fontes et al., 2015), psilocybin (Carhart‐Harris et al., 2012), and LSD (Carhart‐Harris et al., 2016; Müller, Dolder, Schmidt, Liechti, & Borgwardt, 2018), with the degree of reduction in DMN rsFC related to the degree of “ego dissolution” (Carhart‐Harris et al., 2016; Smigielski, Scheidegger, Kometer, & Vollenweider, 2019). Other studies have found that meditative states also elicit decreases in DMN activity and rsFC, especially within the PCC, a finding which may be related to meditation's ability to reduce mind wandering by not getting caught up in “mental chatter” (Brewer et al., 2011; Brewer, Garrison, & Whitfield‐Gabrieli, 2013; Garrison, Scheinost, Constable, & Brewer, 2014; Garrison, Zeffiro, Scheinost, Constable, & Brewer, 2015). In contrast, rumination of negative thoughts (Killingsworth & Gilbert, 2010), as commonly found in depression, appears to be characterized by excessive DMN activity and rsFC (Hamilton, Farmer, Fogelman, & Gotlib, 2015; Sheline et al., 2009; Whitfield‐Gabrieli & Ford, 2012).

Floatation‐REST has been found to have short‐term anxiolytic, antidepressant, and analgesic effects (Bood et al., 2006; Feinstein, Khalsa, Yeh, Wohlrab, et al., 2018; Kjellgren et al., 2001), yet little is known about how the intervention impacts brain networks such as the DMN. Subjectively, the intervention has been found to induce altered states of consciousness, often described as a liminal state, somewhere between being asleep and awake (Kjellgren, Lyden, & Norlander, 2008). Subjects will also frequently describe out‐of‐body experiences (Kjellgren et al., 2008) characterized by difficulty discerning where their outer body begins and where it ends. While Floatation‐REST has sometimes been referred to as a form of sensory deprivation, this term is now considered outdated and a misnomer for the experience (Suedfeld & Coren, 1989). Rather than depriving the senses, we have found that Floatation‐REST actually enhances awareness for internal sensations such as the breath and heartbeat, making the float environment naturally conducive to meditative states (Feinstein, Khalsa, Yeh, Al Zoubi, et al., 2018). Since this was the first fMRI study to examine changes in rsFC related to Floatation‐REST, we utilized an exploratory analytical approach that provided a comprehensive voxel‐wise survey of functional connectivity (FC) across the whole brain using multivariate distance matrix regression (MDMR) (Anderson, 2001; Elliott, Romer, Knodt, & Hariri, 2018; Koyama, O'Connor, Shehzad, & Milham, 2017; Misaki et al., 2018a, 2018b; Satterthwaite et al., 2016; Shehzad et al., 2014; Talukdar, Román, Operskalski, Zwilling, & Barbey, 2018). This data‐driven approach identified clusters showing a significant change in rsFC from pre‐ to post‐float, and these MDMR‐identified clusters were used to guide subsequent seed‐based connectivity analyses. Finally, an active control condition (Chair‐REST) was employed to determine whether the changes in rsFC were specific to floating, or more generally related to any form of REST characterized by: (a) resting wakefulness, (b) lying in a supine position, (c) minimal behavioral output, movement, and speech, and (d) reduced exposure to stimuli from the external environment.

## 2 METHODS

### 2.1 Participants

Fifty‐six healthy adults between 18 and 55 years of age were recruited from a community sample using a subject database maintained at the Laureate Institute for Brain Research. All study procedures were approved by the Western Institutional Review Board (WIRB), and all participants provided their written informed consent prior to participation. Inclusion criteria selected healthy adults who were free of any current or past neurological or psychiatric illness and had no prior experience with Floatation‐REST. Subjects were excluded if they: (a) met criteria for a psychiatric disorder based on the Mini‐International Neuropsychiatric Interview (MINI) version 6.0, (b) failed a urine screen and/or breathalyzer test administered prior to each brain scan to ensure no one was acutely under the influence of any alcohol, drugs, or psychotropic medications (benzodiazepines, opiates, selective serotonin reuptake inhibitors, dopamine agonists, barbiturates, marijuana, MDMA, LSD, psilocybin, and peyote), (c) were pregnant (as detected by a urine test), (d) had noncorrectable vision or hearing problems, (e) had a skin condition or open wound that could cause pain when exposed to saltwater, (f) had any MRI contraindications (e.g., BMI >40 or any metal in the body), or (g) had any prior exposure to Floatation‐REST. Eight subjects in the Floatation‐REST condition were excluded from the final analysis (Figure S1), requiring recruitment of additional float subjects to ensure a matched sample size. The final sample contained 48 subjects with clean and complete datasets (24 Float‐REST subjects and 24 Chair‐REST subjects) and these data are available from the corresponding author upon reasonable request. Participant demographics and baseline functioning are shown in Table 1 for each group.

**TABLE 1:** Participant demographics and baseline measures

	Float‐REST	Chair‐REST
Number of subjects	24	24
Sex (male/female)	11/13	12/12
Age (years)	32.4 (10.6)	30.6 (10.8)
Education (years)	14.6 (2.1)	14.4 (2.0)
General depression (IDAS)	32.6 (4.6)	30.8 (5.2)
Trait anxiety (STAI)	31.4 (6.4)	28.7 (5.6)
Sleepiness pre‐REST MRI (KSS)	3.7 (1.6)	4.0 (1.7)
Sleepiness post‐REST MRI (KSS)	4.3 (1.7)	4.1 (1.9)

*Note*: Both groups were well‐matched, with no significant between‐group differences (*p* >.05 across all variables using Welch's *t*‐test). Values in parentheses represent the standard deviation.

Abbreviations: IDAS, Inventory of Depression and Anxiety Symptoms (Watson et al., 2007); KSS, Karolinska Sleepiness Scale (Kaida et al., 2006); STAI, State‐Trait Anxiety Inventory—Trait Version (Spielberger, 1983).

### 2.2 Procedure

**FIGURE 1:**
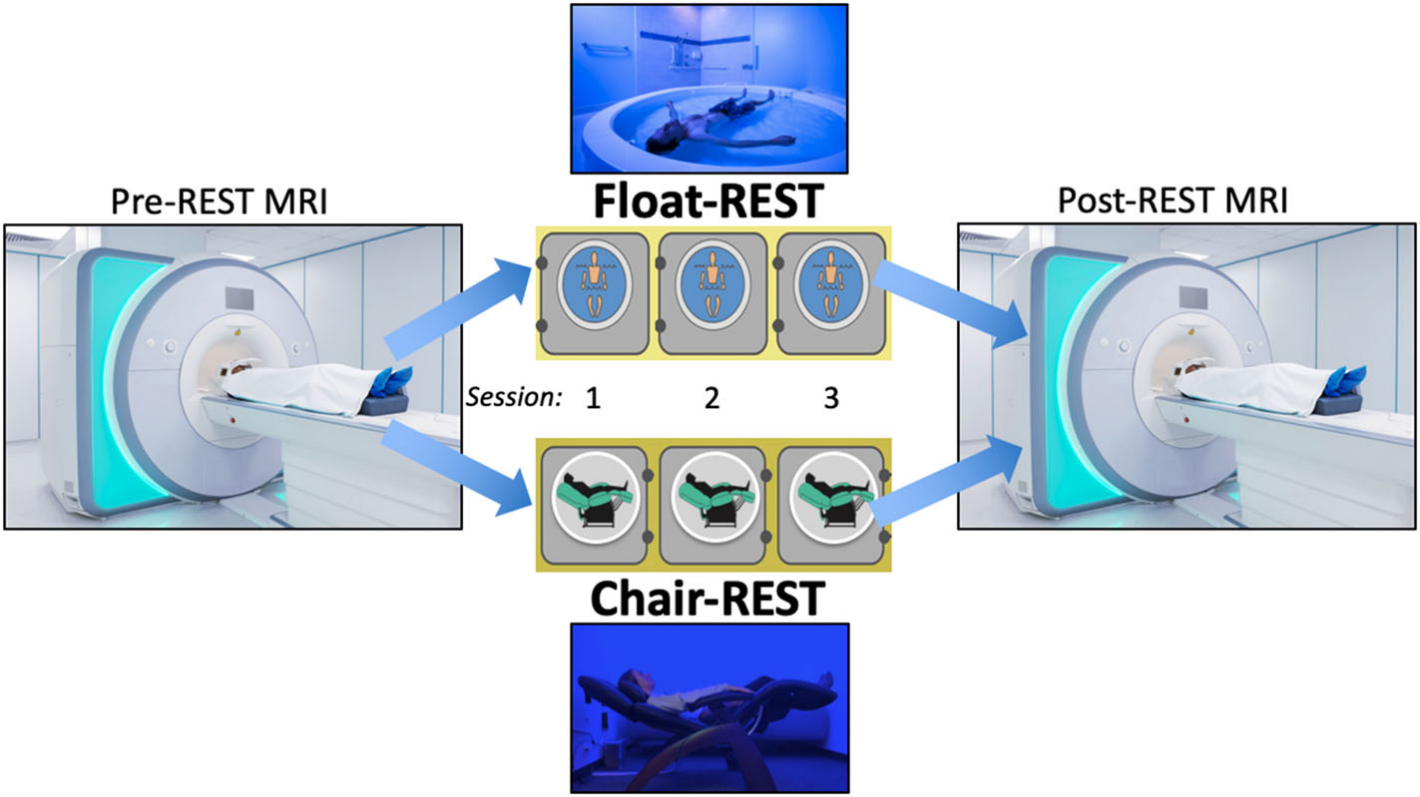
Experimental design. The entire protocol took approximately 1 month for each subject to complete. Participants first underwent a baseline MRI scan (“Pre‐REST MRI”) where they completed an eyes‐open resting state run at the beginning of the scan. Afterward, participants were randomly assigned to complete three 90‐min sessions of either Floatation‐REST (“Float‐REST”; top picture) or the Zero‐Gravity Chair (“Chair‐REST”; bottom picture). All REST sessions were completed over a 3‐week time period, with approximately 1 week between each session. The first two sessions were designed to help acclimate participants to the environment and ensure that everyone could complete a 90‐min session in the dark. Immediately following the third REST session, participants underwent another MRI scan (“Post‐REST MRI”) where they completed a second eyes‐open resting state run at the beginning of the scan

Using a randomized controlled design (Figure 1), participants underwent fMRI before and after completing three sessions of either Floatation‐REST (“Float‐REST”) or the Zero‐Gravity Chair (“Chair‐REST”). Procedures were tailored toward closely matching the two experimental conditions (Float‐REST and Chair‐REST) which both featured 90‐min sessions of reduced light and sound, reduced pressure on the spinal cord while lying supine, and a similar instruction set emphasizing the importance of stillness and wakefulness throughout each REST session (see Supporting Information for more details). After completing the informed consent and all baseline measures (Table 1), participants completed a baseline fMRI scan (Pre‐REST MRI), after which point each participant was randomly assigned to complete three 90‐min sessions of either Float‐REST or Chair‐REST. All REST sessions were completed over a 3‐week time period with ~1 week between each session. For each session, participants were instructed to float with the lights off for 90 min. The selected duration was based on a survey of what most recreational float centers offer at their facilities (Floatation Survey, 2018), which indicated that most healthy participants should be able to safely tolerate 90 min of floating. The first 2 REST sessions were designed to: (a) acclimate participants to the REST environment, (b) reduce the effects of novelty, and (c) ensure that all participants could successfully complete 90 min of REST during their third REST session. Immediately following the third REST session, participants completed another fMRI scan (Post‐REST MRI). To minimize the impact of scanning time, all fMRI scans (Pre‐REST and Post‐REST) occurred at the same time of day in the late afternoon.

In both the Float‐REST and Chair‐REST conditions, a blue LED light remained illuminated in the background and could be turned off by the participant using an air switch. The air switch was linked to a digital clock in the control room, allowing for the automated calculation of the total amount of time that a participant was floating with the lights off. In addition, a microphone in each room provided a real‐time continuous audio feed to a nearby control room, where the experimenter remained throughout the REST session so that they could quickly address any issues that may arise and monitor that the participant remained floating throughout the session. Following 90 min of REST, the experimenter remotely turned on an overhead light in the room to signal that the session was over. In order to standardize instructions, an identical script was read to participants prior to each REST session regardless of whether they were randomized to the Float‐REST or Chair‐REST condition (Supporting Information).

Before and after each REST session, participants rated their current subjective state on the Spielberger State Anxiety Inventory (Spielberger, 1983), a 20‐item questionnaire designed to assess an individual's level of anxiety at the present moment. Participants also completed the Serenity scale on the expanded form of the Positive and Negative Affect Schedule (PANAS‐X), which has participants rate how calm, relaxed, and at ease they feel at the present moment using a 5‐point Likert‐type response scale (Watson & Clark, 1999). State anxiety and serenity scores were converted into the percent of maximum possible (POMP units) for each scale (Cohen, Cohen, Aiken, & West, 1999) and change scores were computed between the pre‐ and post‐REST ratings. In addition, after each REST session, participants rated the overall pleasantness of their REST experience on a 100‐point bipolar valence scale going from 0 (Extremely Unpleasant) to 100 (Extremely Pleasant), with the slider starting in the middle of the scale at 50 (Neutral). After each MRI scan, participants rated their level of sleepiness during the scan using the Karolinska Sleepiness Scale (Kaida et al., 2006).

### 2.3 MRI measurement

Magnetic resonance imaging was conducted using a whole‐body 3 Tesla MR750 MRI scanner (GE Healthcare, Milwaukee, WI) equipped with a 32‐channel receive‐only head array coil (Nova Medical, Wilmington, MA). The resting‐state fMRI scan was the first scan of each session. Subjects were instructed to remain still with their eyes open while looking at a fixation cross on the screen, and to “try to clear your mind and don't think about anything in particular.” Immediately following each resting‐state scan, subjects were asked if they were able to remain awake and keep their eyes open throughout the duration of the scan. A single‐shot gradient‐recalled echo‐planner imaging (EPI) sequence with sensitivity encoding (SENSE) was acquired during the resting‐state scan with the following parameters: TR = 2,000 ms, TE = 27 ms, FA = 40°, FOV = 240 mm, 37 axial slices with 2.9 mm thickness with 0.5 mm gap, matrix = 96 × 96, SENSE acceleration factor R = 2. The EPI images were reconstructed into a 128 × 128 matrix that produced 1.875 × 1.875 × 2.9 mm^3^ voxel volume. Each resting‐state fMRI scan had a total time of 8 min 8 s (244 volumes). As an anatomical reference, T1‐weighted MRI images were acquired using a magnetization‐prepared rapid gradient‐echo (MPRAGE) sequence with the following parameters: FOV = 240 × 192 mm, matrix = 256 × 256, 130 axial slices, slice thickness = 1.1 mm, 0.938 × 0.938 × 1.1 mm3 voxel volume, TR = 5 ms, TE = 1.948 ms, R = 2, flip angle = 8°, delay time = 1,400 ms, inversion time = 725 ms, sampling bandwidth = 31.25 kHz, scan time = 5 min 4 s. Throughout each scan, a pneumatic belt placed around the subject's torso was used to record respiration, and a photoplethysmograph with an infrared emitter placed under the pad of the subject's index finger was used to record pulse oximetry.

### 2.4 MR image processing

Imaging analyses were carried out using the Analysis of Functional NeuroImages (AFNI) software (http://afni.nimh.nih.gov/afni/). The afni_proc.py command was used to preprocess the data using the default parameters unless otherwise noted. The first three volumes were omitted from the analysis. The despike option was applied to replace outlier time points with interpolation. RETROICOR (Glover, Li, & Ress, 2000) and respiration volume per time (RVT) correction (Birn, Smith, Jones, & Bandettini, 2008) were applied to remove cardiac‐ and respiration‐induced noise in the blood oxygenation level‐dependent (BOLD) signal, and spatially smoothed with a Gaussian kernel (FWHM = 6 mm).

Slice‐timing differences were adjusted by aligning to the first slice, and motion correction was applied by aligning all functional volumes to the first volume. EPI volumes were acquired using the 3dvolreg AFNI program with two‐pass registration. The volume with the minimum outlier fraction of the short EPI dataset acquired immediately after the high‐resolution anatomical (MPRAGE) brain image was used as the registration base. Linear warping was applied to the MNI space and resampled to 2 mm^3^ voxels.

Noise reduction was implemented by regressing out: (a) low‐frequency fluctuation from the signal time course (third‐order polynomial model), (b) 12 motion parameters (3 shift and 3 rotation parameters with their temporal derivatives), (c) local white matter average signal (ANATICOR; Jo, Saad, Simmons, Milbury, & Cox, 2010), and (d) three principal components of the ventricle signal from the signal time course. We used FreeSurfer 5.3 (http://surfer.nmr.mgh.harvard.edu/) to extract white matter and ventricle masks from the anatomical image of an individual subject and then warped them to the normalized fMRI image space. Frame‐wise displacement (FD) and DVARS were calculated according to Power, Barnes, Snyder, Schlaggar, & Petersen, 2012 using the FSL motion outliers package. To minimize noise related to movement, subjects were excluded who had a scan with an average FD >0.25 mm or a DVARS >0.5% change in BOLD (Power et al., 2014). In addition, we censored individual time points, and the prior TR whenever the average root mean square (RMS) motion was greater than 0.2 mm.

### 2.5 Multivariate distance matrix regression

As previously mentioned, Floatation‐REST is an intervention that has not yet been explored using fMRI. Thus, we harnessed an exploratory data‐driven approach using MDMR (Elliott et al., 2018; Shehzad et al., 2014) to guide subsequent seed‐based connectivity analyses. MDMR performs a comprehensive voxel‐wise survey of FC changes across the whole brain using a permutation test which minimizes false‐positives and identifies voxels whose whole‐brain connectivity patterns vary significantly according to a prespecified variable. Since the aim of the study was focused on understanding the rsFC changes elicited by Floatation‐REST, the prespecified variable in our MDMR analysis was the difference between the pre‐ and post‐REST scans in the Float‐REST group. Significant MDMR clusters were utilized in subsequent seed‐based analyses examining for significant group × time interactions (using an ANOVA) as well as significant changes within each condition (using paired *t*‐tests). The following steps were applied to the resting‐state fMRI data. First, we downsampled images to 4 mm^3^ voxels after applying an anatomical brain mask to avoid mixing noise from outside the brain. The downsampling stage was necessary to alleviate the computational overheads of the whole‐brain voxel‐wise connectivity matrix. Regression of white matter and the ventricles was not applied during resampling since it was already completed at the preprocessing stage. The data was further reduced by applying a gray matter mask extracted from the MNI152 template brain, reducing the total number of voxels to 18,592 for each subject. In each voxel, a connectivity map from that voxel to all other voxels was made with Pearson's correlations, and the dependent variable was a distance matrix of the connectivity maps between subjects. The MDMR procedure (Shehzad et al., 2014) handles each voxel independently using a single multivariate omnibus statistic, whereas traditional seed‐based analyses encompass many correlation statistics for each voxel. For each voxel, the connectivity map from the voxel's signal time‐course to all other voxels is calculated using Pearson's correlation. From there, the distance of the maps between subject *i* and *j* for each voxel, (*d*_*ij*_) was calculated using Euclidean distance after applying Fisher's *z*‐transformation on the connectivity maps. MDMR constructs a regression model that associates the design matrix Х with predictor variables. Herein, the dissimilarities between connectivity maps across subjects are the predictor variable. The model is evaluated using a pseudo‐*F* value statistic as follows:

$$F=\frac{\frac{\mathrm{tr}\left(\mathrm{HG}\right)}{m-1}}{\frac{\mathrm{tr}\left[\left(I-H\right)G\right]}{n-m}}$$

with

$$G=\mathrm{CAC}\;\text{where}\;C=\left(\;I-\frac{1}{n}\;11^{T}\right)$$

and

$$A={\left(-\frac{1}{2}\;d_{\mathrm{ij}}^{2}\right)}_{1\leq i;j\leq n}$$

In the above formula, *n* is the number of participants, *I* is the *n* × *n* identity matrix, and 1 is a vector of 1's. *C* serves as mean‐centering of the columns and rows of *A*, and *tr* is the matrix trace operator. The design matrix *X* was formed to have one column that represents the group factor encoded as 0's and 1's (e.g., 1's indicates postscans and 0's indicates prescans). Finally, a column of 1's was added for the intercept. In our analysis, we found the design matrix to be rank‐deficient due to collinearity between nuisance variables and subject‐wise regressors. Thus, we solved this issue by applying the singular value decomposition (SVD) on the design matrix (Mandel, 1982). SVD decomposed the design matrix to *X* = *USVT* in which columns of *U* become orthogonal to each other. We rewrite *G* = *Xβ* as, *G* = *USVTβ* = *Uα*. *H* = *UU* ^*T*^. MDMR assesses an individual effect of regressors on a design matrix that excludes the effects of interest columns. We refer to this matrix as the partial design matrix *X*_*N*_. Pseudo‐*F* value of the effect of interest is obtained by

$$F_{I}=\frac{\mathrm{tr}\left(H_{I}G\right)/\left(m_{I}\right)}{\mathrm{tr}\left[\left(I-H\right)G\right]/\left(n-m\right)}$$

With *H*_*I*_ = *H* − *H*_*N*_ and $H_{N}=U_{N}U_{N}^{T}$, $U_{N}S_{N}V_{N}^{T}=\mathrm{SVD}\left(X_{N}\right)$ and *m*_*I*_ is the number of effect of interest regressors. The significance of the pseudo‐*F* value was evaluated by permutation test with 10,000 repeats and thresholded at *p* <.005 voxel‐wise, across the whole brain. Clusters with at least nine adjacent voxels in MDMR space (72 voxels in our original fMRI space) were used as seeds to avoid spurious correlations that can occur with smaller seeds (e.g., Fox, Liu, & Pascual‐Leone, 2013).

### 2.6 Seed‐based FC analysis

Whole‐brain FC analysis was conducted by calculating the Pearson's correlation between the mean time series of each MDMR seed's voxels and the time courses of all other voxels in the brain. Fisher's *r*‐to‐*z* transformation was applied to all correlation coefficients. A group‐by‐time interaction analysis was conducted using a 3‐way analysis of variance implemented in the AFNI program 3dANOVA3. The model option was set to type = 5 in which the group (Float‐REST and Chair‐REST; *df* = 1) and session (pre‐ and post‐REST; *df* = 1) were set as fixed factors, while subjects were set as a random factor (*df* = 47). To reveal brain regions that showed significant within‐subject changes in FC from the pre‐ to post‐REST scans, a paired *t*‐test was applied within each group (Float‐REST and Chair‐REST) using AFNI's 3dttest++ program for each of the 9 MDMR seeds. AFNI's 3dClustSim was applied along with the non‐Gaussian spatial autocorrelation function (ACF) option (Cox, Chen, Glen, Reynolds, and Taylor (2017) to estimate the effective cluster size which minimizes false positives (Eklund, Nichols, & Knutsson, 2016). A permutation test (*n* = 10,000) using the Smith procedure (Winkler, Ridgway, Webster, Smith, & Nichols, 2014), found that an ACF‐corrected cluster requires a minimum of 189 voxels (*NN* = 1) to be deemed significant at *p* < .05 (using an uncorrected voxel‐wise threshold of *p* < .005, a relatively liberal threshold aimed at elucidating the pattern of connectivity changes that contributed to the significant MDMR statistic).

## 3 RESULTS

The two groups (Float‐REST and Chair‐REST) were well matched on age, sex, and education (Table 1). Since subjects were screened to be free of any psychiatric illnesses, both groups had similarly low levels of depression and anxiety, with no significant between‐group differences on any baseline measures (Table 1). All subjects remained awake and alert during the resting‐state fMRI scans, and there were no significant between‐group differences in self‐reported sleepiness during the MRI scans (Table 1). All subjects (in both groups) successfully completed the entire 90‐min session of REST prior to their post‐REST MRI scan. Moreover, all subjects remained in the dark with the lights out throughout their third REST session with the exception of 4 subjects in the float condition who had a dim blue LED light on for a small portion of the REST session (notably, these 4 subjects were still in the dark for an average of 69 min; range = 53–86 min/session). Consistent with prior studies (Feinstein, Khalsa, Yeh, Al Zoubi, et al., 2018; Feinstein, Khalsa, Yeh, Wohlrab, et al., 2018), Float‐REST induced increases in serenity (mean change ± *SD* during third Float‐REST session = 20.14 ± 19.34; third Chair‐REST session = 3.13 ± 17.86) and decreases in state anxiety (mean change ± *SD* during third Float‐REST session = −7.22 ± 9.45; third Chair‐REST session = 1.81 ± 10.74) that were significantly greater than those elicited by Chair‐REST (Serenity: *t*_46_ *=* 3.17, *p <*.005; State Anxiety: *t*_46_ *= −*3.09, *p <*.005). Subjects also found Float‐REST to be significantly more pleasant than Chair‐REST (*t*_46_ *=* 3.02, *p <*.005; mean pleasantness ± *SD* of third Float‐REST session = 82.67 ± 11.27; third Chair‐REST session = 69.54 ± 18.08).

**FIGURE 2:**
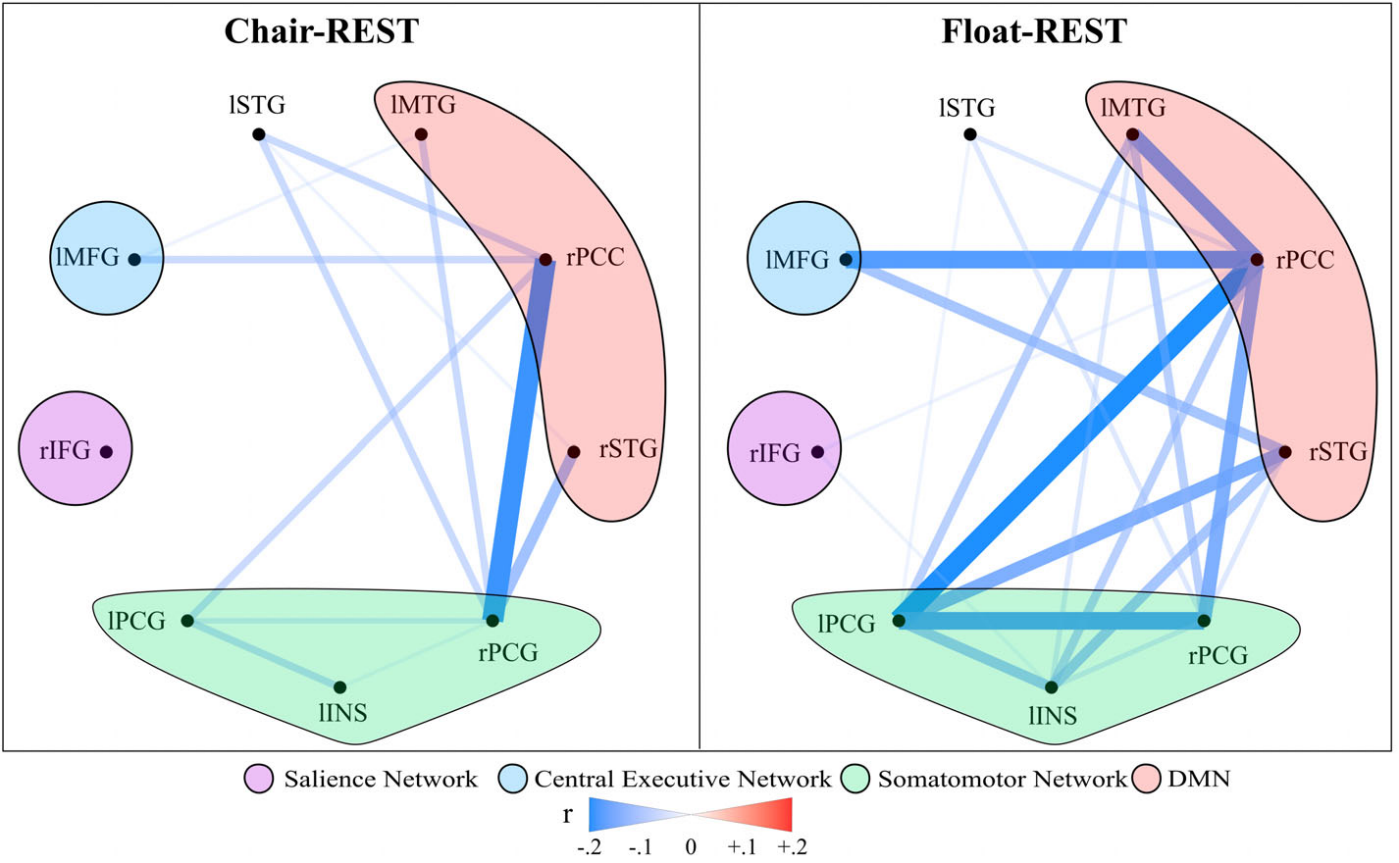
Mean FC change from pre‐REST to post‐REST for each group across the nine MDMR seeds. Both groups exhibited post‐REST reductions in rsFC between the seed regions, with the Float‐REST group showing comparably larger reductions in rsFC, especially within and between the DMN and somatomotor seeds

The MDMR whole‐brain searchlight approach discovered nine clusters whose connectivity pattern varied significantly from the pre‐ to post‐float scan (Table 2). The clusters spanned different networks of the brain but were largely concentrated within the posterior DMN and somatomotor networks. When these clusters were subsequently applied as seed regions in a group‐by‐time interaction analysis using AFNI's 3dANOVA3, there were no connected clusters that survived ACF‐correction, highlighting a lack of any significant between‐group differences. Overall, both groups showed a consistent pattern of reduced rsFC across the 9 MDMR seeds when comparing the post‐REST scan to the pre‐REST scan, with the Float‐REST group showing the largest rsFC reductions (Figure 2).

**TABLE 2:** Location of MDMR seeds

#Voxels	*x*	*y*	*z*	Region	Network	Pseudo *F*‐stat
176	46	−53	12	Right sup. temporal gyrus (rSTG)	DMN	2.9
144	−51	−13	32	Left postcentral gyrus (lPCG)	Somatomotor	3.8
112	−59	−20	1	Left sup. temporal gyrus (lSTG)		3.1
112	−51	16	29	Left middle frontal gyrus (lMFG)	Executive	2.7
104	−42	−66	16	Left middle temporal gyrus (lMTG)	DMN	2.8
96	6	−66	16	Right posterior cingulate (rPCC)	DMN	3.3
80	55	22	3	Right inferior frontal gyrus (rIFG)	Salience	2.7
72	−34	11	11	Left posterior insula (lINS)	Somatomotor	3.5
72	34	38	52	Right postcentral gyrus (rPCG)	Somatomotor	2.8

**FIGURE 3:**
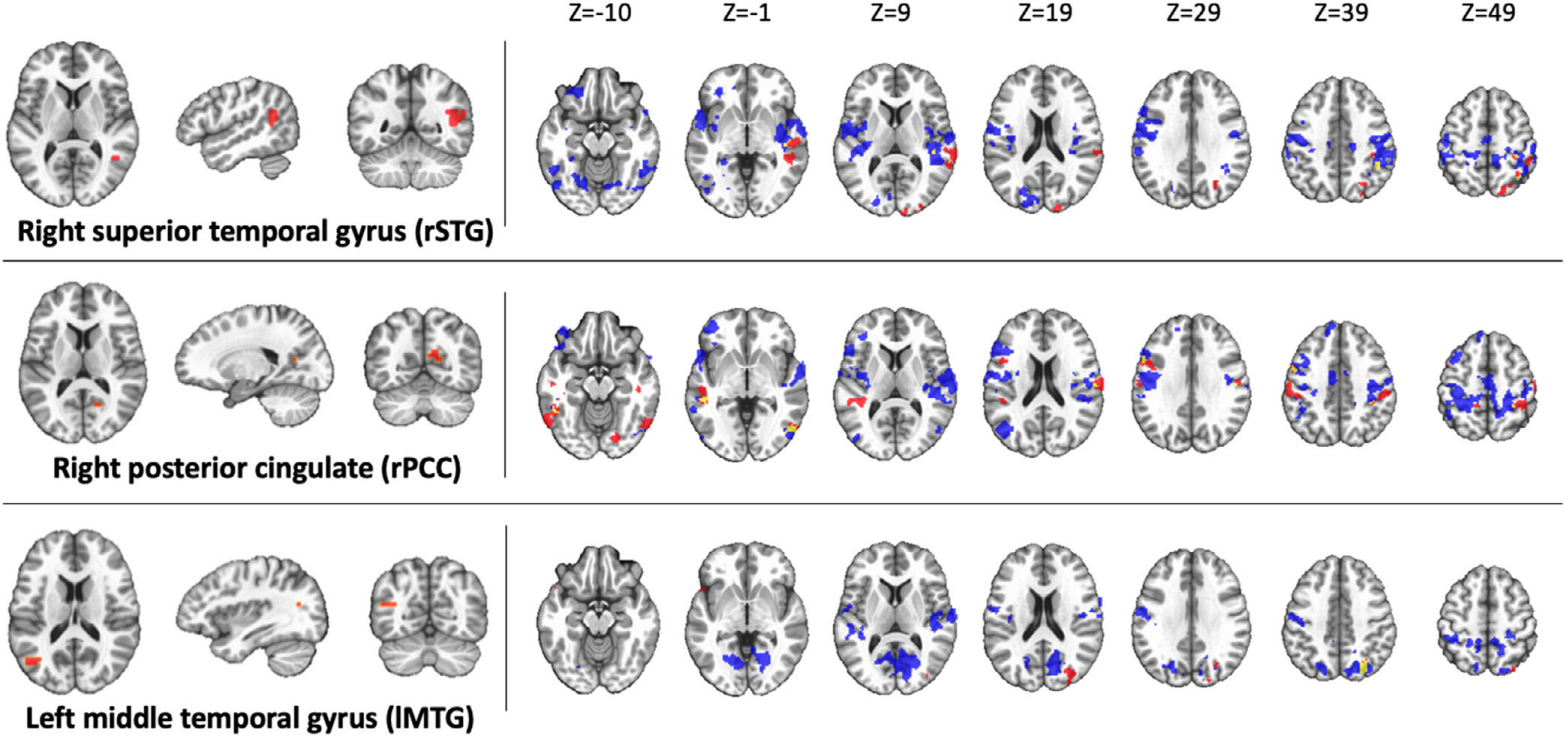
DMN seeds. Regions of significant rsFC change from pre‐REST to post‐REST for each group (blue = Float‐REST; red = Chair‐REST; yellow = overlap) across the 3 DMN MDMR seeds shown on the left side of the panel. All clusters shown survived ACF‐correction (*p* <.05) and signify overall decreases in rsFC during the post‐REST scan. Clusters are displayed using neurological convention (i.e., right side of image corresponds to the right hemisphere)

**FIGURE 4:**
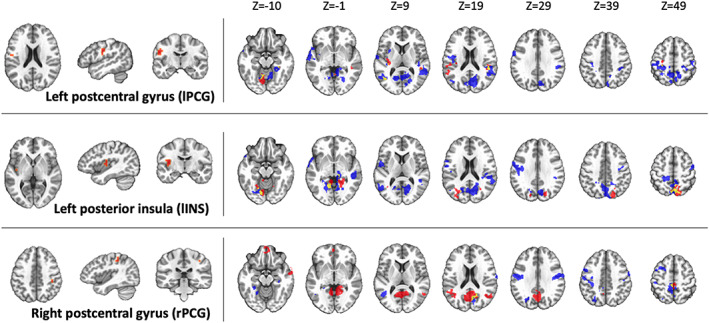
Somatomotor seeds. Regions of significant rsFC change from pre‐REST to post‐REST for each group (blue = Float‐REST; red = Chair‐REST; yellow = overlap) across the 3 somatomotor MDMR seeds shown on the left side of the panel. All clusters shown survived ACF‐correction (*p* <.05) and signify overall decreases in rsFC during the post‐REST scan. Clusters are displayed using neurological convention (i.e., right side of image corresponds to the right hemisphere)

**FIGURE 5:**
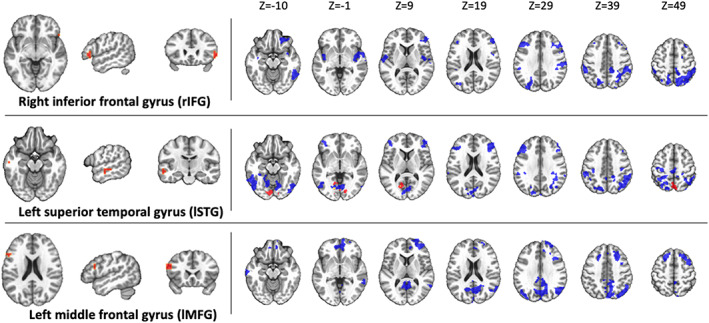
Other seed regions. Regions of significant rsFC change from pre‐REST to post‐REST for each group (blue = Float‐REST; red = Chair‐REST; yellow = overlap) across the 3 final MDMR seeds shown on the left side of the panel. All clusters shown survived ACF‐correction (*p* <.05) and signify overall decreases in rsFC during the post‐REST scan. The top seed (rIFG) is a hub of the salience network, and the bottom seed (lMFG) is a hub of the central executive network. Clusters are displayed using neurological convention (i.e., right side of image corresponds to the right hemisphere)

Subsequent analyses applied the same MDMR seeds to each group separately to examine for within‐subject changes in rsFC from the pre‐ to post‐REST scan. The Float‐REST group had a total of 71 significant clusters across the nine seed regions that survived ACF‐correction (Figures 3, 4, 5), with an average cluster size of 962 voxels (2 mm^3^ voxels). In contrast, the Chair‐REST group showed a much sparser pattern with about half as many significant clusters (37 in total) that were also smaller in size (average cluster size of 416 voxels). Once again, across all significant clusters, rsFC decreased from the pre‐ to post‐REST scan. In fact, there was not a single significant association, in either condition (Float‐REST and Chair‐REST), which showed a pre‐ to post‐REST increase in rsFC.

The Float‐REST group exhibited a consistent pattern of reduced rsFC within and between posterior hubs of the DMN, especially posterior cingulate and precuneus, and throughout a large swath of cortical tissue encompassing somatomotor cortices (Figures 3, 4, 5), including the premotor cortex, primary and secondary somatosensory cortices, extending into the posterior insula, as well as supramarginal gyrus and paracentral lobule. Float‐REST also showed significant rsFC reductions between regions of the salience and central executive networks and their connections with DMN and somatomotor hubs (Figure 5). In contrast, the Chair‐REST group did not show this same pattern of significant connectivity changes, and for most of the MDMR seeds there were few (if any) DMN or somatomotor clusters that survived ACF‐correction (Figures 3, 4, 5). In general, there was very little overlap between Float‐REST and Chair‐REST clusters that survived ACF‐correction. The only substantial overlap between conditions was found in connectivity patterns between: (a) the left posterior insula seed and precuneus and (b) the right postcentral gyrus seed and the right PCC (Figure 4).

## 4 DISCUSSION

This study represents the first resting‐state functional neuroimaging investigation of Floatation‐REST, a unique method for systematically reducing stimulation of the human nervous system. The resting‐state fMRI data was analyzed using MDMR, a statistically‐stringent whole‐brain searchlight approach aimed at finding peak clusters of connectivity change between the pre‐ and post‐float brain scans. The results revealed that a 90‐min session of Float‐REST elicited a consistent pattern of decreased rsFC within and between posterior hubs of the DMN (the posterior cingulate and precuneus, and its temporoparietal flanks) and a large swath of cortical tissue centered around the somatomotor network, including primary and secondary somatosensory cortices extending into the posterior insula. Together, this network of somatosensory tissue creates a moment‐to‐moment mapping of the current state of the body, being the primary cortical recipient of afferent sensory information arising from the surface of the skin all the way down to the visceral organs within the body (Craig, 2002; Damasio, 1999).

The post‐float reduction in rsFC observed between posterior hubs of the DMN and the somatosensory cortices suggest that Floatation‐REST may reduce self‐reflective processes directed toward the current state of the body. Such an interpretation is consistent with prior research showing a critical role of the DMN in creating a sense of self (Qin & Northoff, 2011), in addition to other research showing that posterior hubs of the DMN can facilitate reductions in conscious awareness for somatosensory stimulation (Boly et al., 2007). Indeed, there are multiple maps of the body contained within parietal cortices (Berlucchi & Aglioti, 2010; Longo, Azañón, & Haggard, 2010) that contribute to self‐awareness. For example, recent work has discovered a causal role for posteromedial cortices in dissociation (Vesuna et al., 2020). The temporoparietal junction has been shown to be critical for the multisensory integration of visual, tactile, and vestibular signals that create our sense of the body in space, and when disturbed, can lead to out‐of‐body experiences (Ionta et al., 2011). The FC between temporoparietal junction and posterior insula (Ionta, Martuzzi, Salomon, & Blanke, 2014) is rapidly altered by shifts in self‐location (i.e., *the experience of where I am in the world*) and first‐person perspective (i.e., *the experience of where I perceive the world from*), and the posterior parieto‐insular cortex is particularly involved in processing vestibular sensations (Eickhoff, Weiss, Amunts, Fink, & Zilles, 2006). The posterior insula is closely connected with both the somatosensory cortices and the posterior cingulate (Cauda et al., 2011; Deen, Pitskel, & Pelphrey, 2011), creating a strong interconnected system between hubs of the posterior DMN and the somatosensory cortices. Since Floatation‐REST has been shown to significantly reduce muscle tension (Feinstein, Khalsa, Yeh, Al Zoubi, et al., 2018; Kjellgren et al., 2001), it may be possible that the intervention directly alters the representation of this tension within the brain's body maps. This is consistent with a prior fMRI study that found reduced activity in the posterior cingulate, somatomotor, and insular cortices following progressive muscle relaxation (Kobayashi & Koitabashi, 2016). In an exploratory correlation analysis, we found that the greater the decrease in connectivity between posterior insula and somatosensory cortices, the greater the serenity induced by the float experience (Figure S2), highlighting a potentially important role for these somatosensory rsFC changes in eliciting subjective relaxation.

With the exception of somatosensory cortices, the observed post‐REST decreases in rsFC largely did not coincide with the other senses reduced by REST (e.g., primary and secondary visual and auditory cortices did not show significant changes in rsFC). Instead, the changes found in our study were largely concentrated within higher‐level association cortices such as the posterior hubs of the DMN. Notably, a preserved DMN has been found in individuals who are congenitally blind suggesting that “the absence of a particular sensory modality does not qualitatively affect default functionality” (Burton, Snyder, & Raichle, 2014). There is even evidence of a DMN in young children and babies who are in utero (van den Heuvel & Thomason, 2016), providing further confirmation that DMN functionality does not require exposure to exteroceptive sensory information. The present study extends this notion even further by showing that DMN rsFC, which is reflexively heightened during states of resting wakefulness (Raichle, 2015; Raichle et al., 2001), appears to be significantly reduced after a prolonged period of REST.

In contrast to the posterior DMN, there was comparatively little change found in the anterior DMN following Floatation‐REST. This was somewhat surprising given the well‐known role of the mPFC in self‐related processing (Gusnard, Akbudak, Shulman, & Raichle, 2001), especially self‐referential thought (Qin & Northoff, 2011) and rumination (Hamilton et al., 2015). Prior work from our lab has shown that the mPFC was not necessary for self‐awareness in a rare lesion patient who had bilateral mPFC damage but preserved rsFC throughout his posterior DMN (Philippi et al., 2012). The only evidence of significant mPFC involvement in the present study was found in relation to the lMFG seed near the left dorsolateral prefrontal cortex (Figure 5). This region of central executive network is often the target of transcranial magnetic stimulation (TMS) for the treatment of depression, an intervention that also appears to impact the mPFC (Liston et al., 2014). Since our study only employed healthy subjects, future studies will have to assess whether similar rsFC reductions in mPFC emerge in depressed patients following Floatation‐REST.

When examining the same MDMR seeds in subjects randomized to the Chair‐REST condition, reduced rsFC was also observed, but there were no significant group by time interactions with Float‐REST. However, when comparing the degree of rsFC change, it became evident that the Chair‐REST condition did not elicit as robust of a reduction in rsFC as the Float‐REST condition (Figure 2). Moreover, the vast majority of DMN and somatomotor clusters that survived ACF‐correction in the Float‐REST group failed to surpass significance in the Chair‐REST group (Figures 3, 4, 5). Thus, it remains possible that the added degree of weightlessness and loss of proprioception conferred by Floatation‐REST may have preferentially impacted the brain's body maps. Nevertheless, the lack of significant between‐group differences suggests that reduced stimulation of the nervous system via any form of REST may be reflected by reduced rsFC within the brain networks most responsible for creating and mapping our sense of self.

While both the Float‐REST and Chair‐REST conditions shared many overlapping features (90‐min sessions, supine position, reduced pressure on the spinal cord, being alone in a quiet and dark room, and the same instruction set emphasizing the need for stillness and staying awake), there were some notable differences. Firstly, the sensory reduction (e.g., light, sound, and proprioception) during the Chair‐REST condition was not as complete as during Floatation‐REST; the room housing the zero‐gravity chair was not constructed to eliminate all outside light and sound, and the chair itself, even when reclined, still exerted some degree of pressure on the body and spinal cord. Secondly, the Chair‐REST condition did not entail any exposure to water or salt. Thirdly, participants remained clothed throughout Chair‐REST (whereas individuals were naked during Float‐REST). And finally, the temperature during the Chair‐REST condition was not matched to skin temperature (~95.0 °F), but instead was maintained at a normal room temperature (~73.0 °F). Thus, while the zero‐gravity chair condition contains many active ingredients of REST, the experience was not as immersive as floating in the pool, and the reduction in sensory stimulation was not nearly as complete and all‐encompassing as Floatation‐REST. In addition, the subjective changes elicited by the Chair‐REST condition were not as robust as those elicited by Float‐REST (which participants found to be significantly more pleasurable), and consequently, the Float‐REST condition induced significantly greater increases in serenity and decreases in state anxiety. If future studies could find a REST intervention capable of eliciting similar subjective changes as those induced by Float‐REST, then it may be possible to devise an intervention that would be easier to implement and disseminate.

There are several limitations that should be mentioned. Since this was the first functional neuroimaging study to examine the effects of Floatation‐REST, it will be necessary for future work to confirm these findings. It should be acknowledged that there were several REST sessions and several weeks between brain scans, and these intervening variables could have also contributed to the findings. Even though the groups were well‐matched, the lack of significant between‐group differences in rsFC changes could be due to a number of factors. Since the control condition was an active form of REST, it appears to have elicited a similar pattern of reduced rsFC, making it difficult to discern significant differences. Moreover, with our moderate sample size of 24 subjects in each group (each scanned twice), we were likely underpowered to find a significant group by time interaction. Recent evidence has suggested that the duration of the resting state scan employed in the current study may be insufficient to provide high levels of test–retest reliability (Noble, Scheinost, & Constable, 2019), and therefore, it is possible that measurement variability contributed to the lack of significant differences found between conditions. Finally, it is worth noting that the post‐REST brain scans always occurred after the completion of REST, so no inferences can be made about neural activity changes that occurred during the actual time period of reduced sensory and motor stimulation.

## CONFLICT OF INTEREST

The authors declare no conflict of interest.

## Supporting information


**Appendix** **S1:** Supporting Information

## Data Availability

The final sample contained 48 subjects with clean and complete datasets (24 Float‐REST subjects and 24 Chair‐REST subjects) and these data are available from the corresponding author upon reasonable request.
