# Supplementary material for: Taking the body off the mind: Decreased functional connectivity between somatomotor and default‐mode networks following Floatation‐REST
Source: Hum Brain Mapp. 2021 Apr 9;42(10):3216–27. doi: 10.1002/hbm.25429 (PMC8193533; doi:10.1002/hbm.25429)
Supplement: Supplementary file 1 — Appendix S1: Supporting Information [file HBM-42-3216-s001.docx]

**Supporting Information**

Procedures were tailored toward closely matching the two experimental conditions (Float-REST and Chair-REST). During the informed consent process all participants were read the following script: “*Throughout the day, our brain and body are constantly bombarded by sensory information from the external world. In this study, we aim to understand what happens when you get a chance to disconnect from this constant stimulation by floating in an environment with reduced levels of light and sound, and reduced pressure on the spinal cord*.” Participants were then told that they would be randomized to one of two float conditions, either the “float pool” or the “float chair”, and that both conditions featured reduced light and sound, and reduced pressure on the spinal cord. Prior to each float session, the following script was read to participants regardless of whether they were randomized to the Float-REST or Chair-REST condition: “*While floating, try your best to remain still with the lights off for 90 minutes. You have complete control throughout the experience and can stop at any time. During your float, try not to fall asleep as our study is focused on what happens to the brain while you are awake. Our goal is to scan your brain after floating for 90 minutes, so it is really important that you float for the entire 90 minutes. After the time is up, we’ll turn on the lights to let you know that the float is over.*” No additional instructions were provided for how participants should spend their time during the REST session.

## Floatation-REST condition

All sessions of Floatation-REST occurred in an open circular fiberglass float pool (Figure 1) that was custom-designed for research purposes by Floataway (Norfolk, United Kingdom). The open circular float pool was 8 feet in diameter and contained 11 inches of reverse osmosis water saturated with ~1,800 pounds of USP grade Epsom salt (magnesium sulfate), creating a dense salt water solution maintained at a specific gravity of ~1.26, which allows participants to effortlessly float on their back without having to move any muscles. A shower located next to the float pool was used to wash off all the salt after each session. Since the float pool had no enclosure, the room built around the pool was constructed to be waterproof, soundproof, lightproof, and temperature-controlled. Silent heaters were placed under the pool to maintain the water at a constant temperature and a dedicated heating, ventilation, and air conditioning system maintained the room air at a constant temperature. The temperature of the water and air approximated the surface temperature of the skin (~95.0°F), and could be adjusted remotely by the experimenter in a nearby control room.

The float pool and surrounding room were specially engineered to minimize all sensory signals from visual, auditory, olfactory, gustatory, thermal, tactile, vestibular, gravitational and proprioceptive channels. Visual stimulation was minimized by building an entry door and gasket system which expunged all sources of outside light. In addition, there were no windows inside the float room, and the adjacent room contained a private bathroom that also had no windows, and no lights (which were automatically shut off during the float itself). Thus, when the entry door to the float room was sealed and the blue LED light inside the pool was turned off, the float room was completely dark. Auditory stimulation was minimized by constructing the float room using multiple layers of sound dampening walls with thick insulation and added soundproofing material, restricting most outside airborne sound from entering the room. Structural sounds transmitted via vibrations in the floor were minimized by having the float pool rest on a bed of 48 butyl rubber springs, effectively isolating the pool from the building and preventing structure-borne noises from entering the water. Olfactory stimulation was minimized by using only unscented cleaning products and having the participant shower beforehand to help remove body odors. In addition, the water disinfection system used a combination of ultraviolet light and 35% hydrogen peroxide which does not emit any odors during the oxidative process. Gustatory stimulation was minimized by having participants eat several hours before the float while refraining from eating and drinking during the float. Thermal stimulation was minimized by setting the temperature of the water and the air to closely match the temperature at the surface of the skin, which is typically a few degrees cooler than core body temperature. All temperature sensors were calibrated using a Thermoworks precision thermometer (Utah, USA) certified by the National Institute of Standards and Technology (NIST). Throughout each float session, the water temperature was maintained at 95.0°F (±0.3°F) and the air temperature at the rim of the pool was maintained at 93.0°F (±1.0°F), slightly lower than the water temperature based on the relative humidity in the air. This temperature setting helped minimize the need for thermoregulation while reducing the perceptual boundary between air, body, and water. The specific gravity of the water was calibrated using an H-B Instrument Polycarbonate Hydrometer (Pennsylvania, USA), with a specific gravity range of 1.20-1.42 and NIST calibrated to achieve accuracy within 0.002. The density of the water and salt concentration was maintained at a specific gravity between 1.24-1.26 for all float sessions. The body’s immersion in this dense saline solution minimized stimulation from tactile, vestibular, gravitational, and proprioceptive channels by buffering the body against the forces of gravity and allowing the individual to effortlessly float on their back in a state of stillness.

## Chair-REST condition

Participants randomized to the active control condition remained reclined in a supine position for 90 minutes while lying on a zero-gravity chair (Human Touch Perfect Chair PC510, Classic Power, Series 2) that was located inside a dark and quiet room. The chair (Figure 1) was ergonomically designed to take pressure off the spinal cord and contained memory foam backing to help the chair conform to each participant’s body shape. The room temperature was maintained at 73.0°F (±1.0°F) and all subjects wore clothing while reclined in the chair. In order to match the instruction set across both conditions, the zero-gravity chair was explicitly referred to as the “Float chair” and the act of lying in the chair was referred to as “floating”.

**Supplementary Figure 1: CONSORT flow diagram**.


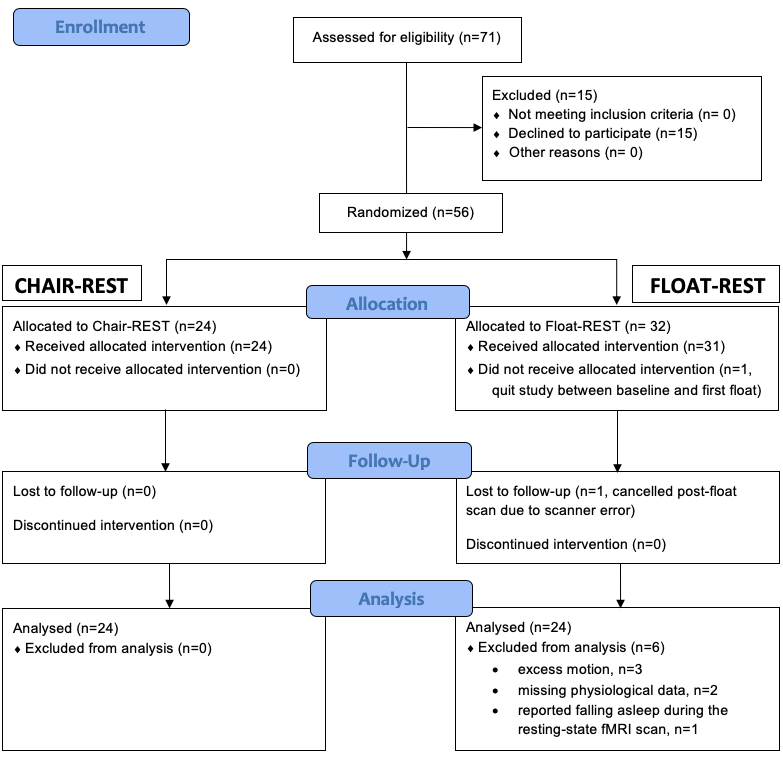


**Supplementary Figure 2: Correlations with subjective measures during Floatation-REST**. (A) Floatation-induced changes in state anxiety and serenity, as well as sleepiness levels during the MRI scan, were correlated with each float subject’s rsFC change from pre-REST to post-REST across the 9 MDMR seeds (see blue lines in Figure 2) using Pearson’s correlation coefficient. The only significant correlations were found between (B) serenity and rsFC changes in the right somatosensory cortex (rPCG) and left posterior insula (lINS), and (C) serenity and rsFC changes in the right somatosensory cortex (rPCG) and left somatosensory cortex (lPCG).

(A)


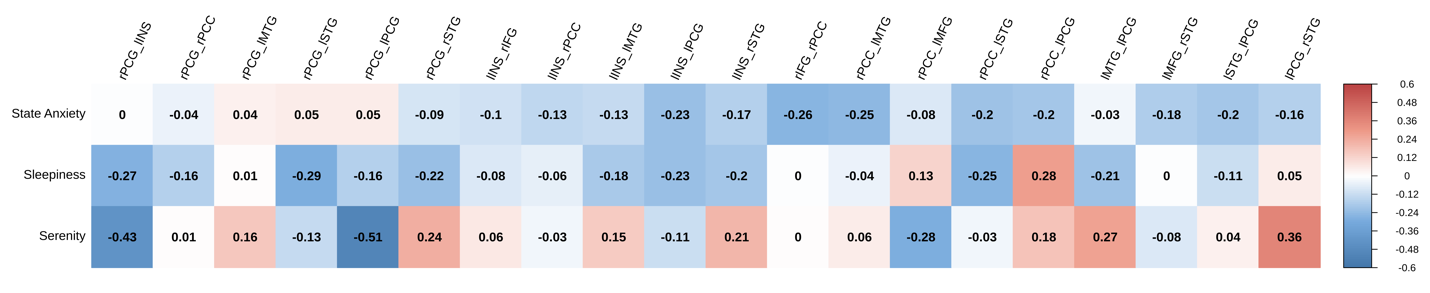


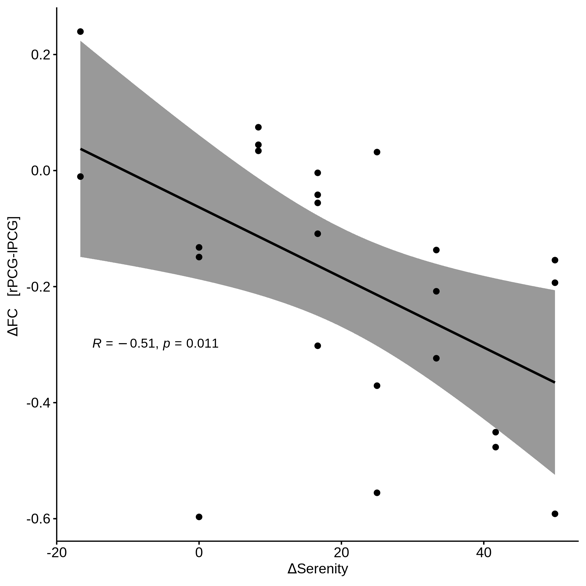

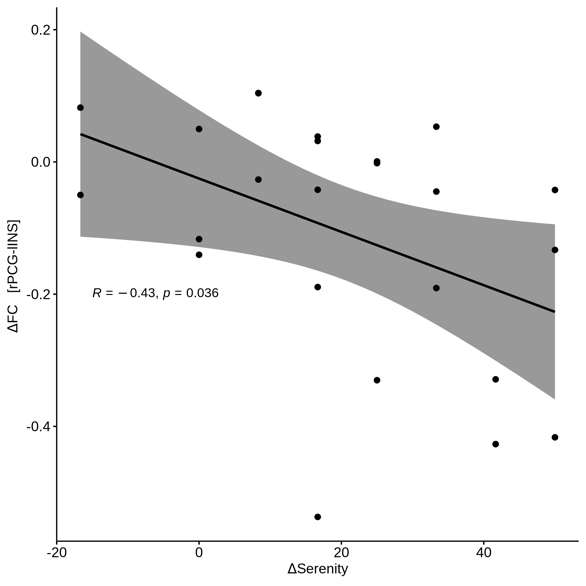
(B) (C)
